# Molecular Dynamics Simulations of Sonic Hedgehog-Receptor and Inhibitor Complexes and Their Applications for Potential Anticancer Agent Discovery

**DOI:** 10.1371/journal.pone.0068271

**Published:** 2013-07-31

**Authors:** Swan Hwang, Sundarapandian Thangapandian, Keun Woo Lee

**Affiliations:** Division of Applied Life Science (BK21 Program), Systems and Synthetic Agrobiotech Center (SSAC), Plant Molecular Biology and Biotechnology Research Center (PMBBRC), Research Institute of Natural Science (RINS), Gyeongsang National University (GNU), Jinju, Republic of Korea; Wake Forest University, United States of America

## Abstract

The sonic hedgehog (Shh) signaling pathway is necessary for a variety of development and differentiation during embryogenesis as well as maintenance and renascence of diverse adult tissues. However, an abnormal activation of the signaling pathway is related to various cancers. In this pathway, the Shh signaling transduction is facilitated by binding of Shh to its receptor protein, Ptch. In this study, we modeled the 3D structure of functionally important key loop peptides of Ptch based on homologous proteins. Using this loop model, the molecular interactions between the structural components present in the pseudo-active site of Shh and key residues of Ptch was investigated in atomic level through molecular dynamics (MD) simulations. For the purpose of developing inhibitor candidates of the Shh signaling pathway, the Shh pseudo-active site of this interface region was selected as a target to block the direct binding between Shh and Ptch. Two different structure-based pharmacophore models were generated considering the key loop of Ptch and known inhibitor-induced conformational changes of the Shh through MD simulations. Finally two hit compounds were retrieved through a series of virtual screening combined with molecular docking simulations and we propose two hit compounds as potential inhibitory lead candidates to block the Shh signaling pathway based on their strong interactions to receptor or inhibitor induced conformations of the Shh.

## Introduction

The sonic hedgehog (Shh) signaling pathway is critical for embryonic development and differentiation, patterning of several tissues, and stem cell renewal [1], [2]. Activity of the Shh signaling is important for maintenance and improvement of coronary vasculature, and proliferation of stem cells in adult, but reversely the aberrant reactivation leads to basal cell carcinoma, myeloid leukemia, and rhabdomyosarcoma [3]–[6]. Additionally, this pathway is related to carcinomas of foregut such as esophagus, stomach, pancreas, and biliary tract as well as small lung cancer and neoplasia of prostate [7]. Shh can spread as a morphogen from its source cell to neighboring cells or up to the maximum 30-cell diameters [8]. Shh undergoes the autocleavage process and subsequently a cholesterol added to C terminus of 20 kDa Shh N-terminal signaling domain (ShhN) and 25 kDa Shh C-terminal signaling domain (ShhC) to be separated, and palmitoylation occurs at N-terminus of ShhN [9]–[11]. These autoprocessing and lipid modification are probably responsible to strict membrane-association of the Shh [12].

The Shh signaling pathway is initiated by cohesion of Shh to its receptor *12-transmembrane cell surface receptor Patched* (Ptch). In absence of the Shh, *G Protein-Coupled Receptor (GPCR)-like protein Smoothened* (Smo) protein resides in cell membrane is fastened by the Ptch and this precludes the signal transduction, but inversely in the case of the ligation of Shh to the Ptch, the Smo to be alleviated. The relieved Smo migrates to the primary cilium and triggers the intracelluar cascade by recruiting downstream components into the primary cilium. Subsequently, the signal transmission accelerates a modification of repressor forms of *Glioma-associated Oncogene Homologue Zinc Finger Protein* (Gli) to its activator forms. The activator forms of Gli move to the nucleus and activate the transcription of the target genes such as *Ptch1*, *Wnt*, *Cyclin D2*, *Plakoglobin* [2], [5], [13].

Shh is structurally homologous to bacterial carboxypeptidase and it contains an obvious catalytic site that presumably possesses a hydrolytic activity. But the evidence of unrelatedness between the surmisable hydrolytic activity and triggering of the Shh signaling pathway was suggested by conducting a variety of mutations at the presumed catalytic residues [14]. A direct binding of the Shh to Ptch, alternatively, was accounted for signal transduction of the Shh signaling pathway through the measurement of its binding affinity according to alteration of the catalytic residues of the Shh.

A disturbance of the Shh signaling pathway by blocking the binding of Shh and Ptch can lead to a cure for multiple cancers. Several macromolecules such as *Cell adhesion molecule-related/down-regulated by oncogenes* (Cdo), *Brother of Cdo* (Boc), *Hedgehog-interacting protein* (Hhip) and the murine:human chimeric 5E1 that lead to disturbance of the Shh signaling pathway by binding to the similar surface of the Shh were reported [15]–[17]. Until recently, a small molecule inhibitor discovered for targeting the Shh itself is robotnikinin that has a portion of 12-membered macrocyclic scaffold and has been formulated to suppress the Shh signaling transduction [18].

We hypothesized that an interruption to the direct binding between the Shh and Ptch by designing a small molecule that binds the Shh pseudo-active site with high affinity can be a valuable expedient to block the Shh signal transduction. In this interaction, an extracellular loop between transmembrane 7 and 8 of Ptch (Ptch loop 2-like loop (PL2)) is the key structural component for the Shh signaling transduction [19], [20].

Contrary to previous study that has performed to discover potential inhibitor candidates for Shh using 3D structural information of robotnikinin and its analogues [21], we devised a scheme for designing potent structure-based chemical lead candidates using representative structures obtained from the dynamic simulations of the Shh-PL2 and -robotnikinin complexes. The 3D structure of Ptch has not solved yet and therefore the PL2 was modeled using Hhip loop 2 (Hhip L2) structure as a template on the grounds that the amino acid sequences of the PL2 are homologous to the Hhip L2 [15]. Additionally, we verified the critical role of the metal ions present in the Shh pseudo-active site using MD simulations of Shh-PL2 with different compositions of the metal ions. Crystallographically determined complex structure of the Shh and robotnikinin is also not available and thus a binding mode of robotnikinin was predicted using protein-ligand docking simulations. Two structure-based pharmacophore models were generated and used in database screening in order to identify the chemical leads bind the pseudo-active site of Shh with high affinity. Two hits mapping the pharmacophoric features of both the pharmacophore models were selected and reported as the potential chemical lead candidates to be used in future Shh-signaling pathway inhibitor design.

## Materials and Methods

### Receptor loop modeling and structural refinement

**Figure 1:**
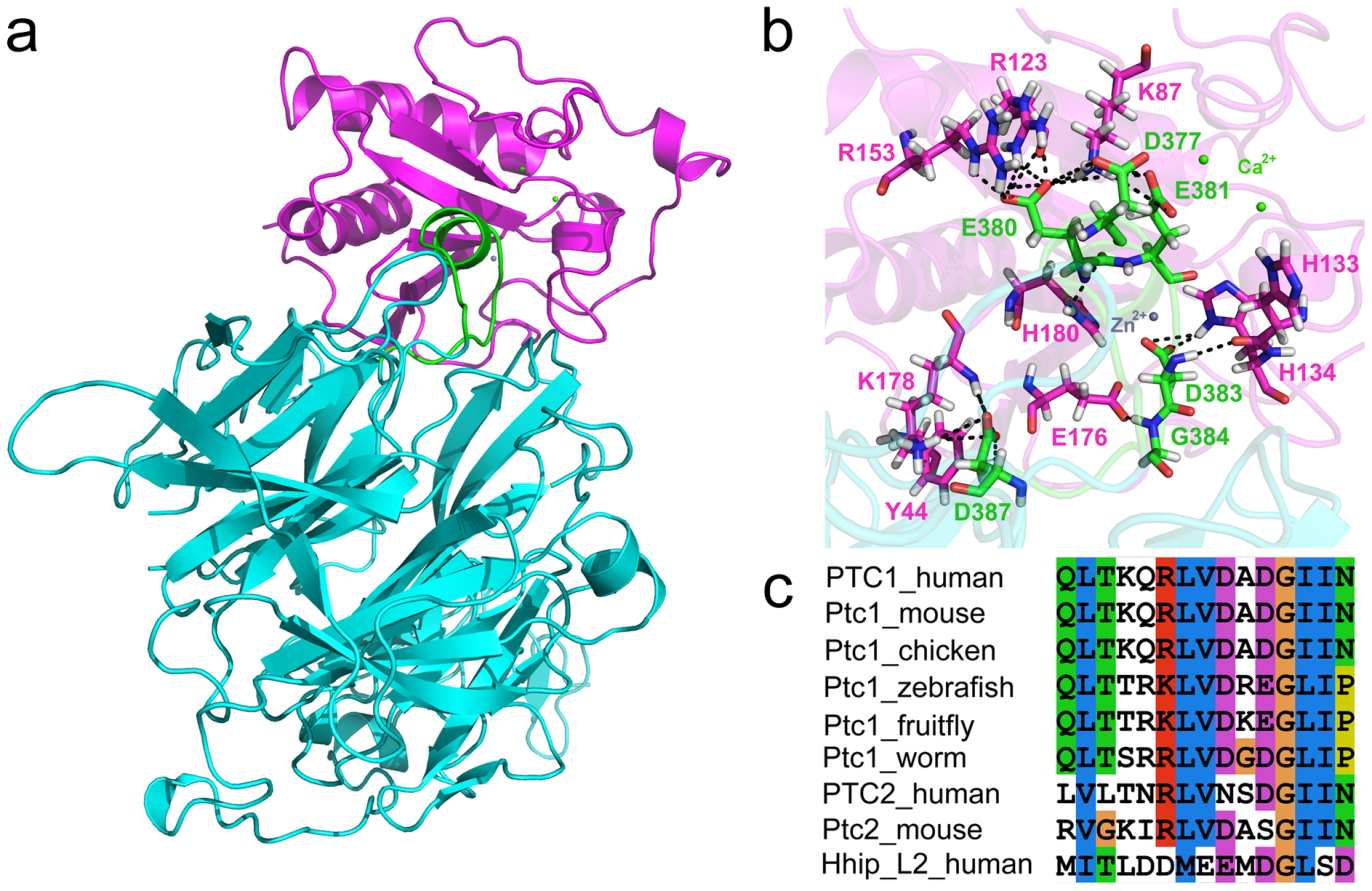
Complex structure of Shh-Hhip and sequence analysis of Hhip L2 with various species of Ptchs. (a) The crystal structure of human Shh-Hhip complex (PDB ID: 3HO5). Shh, Hhip, and Hhip L2 (M373-D387) are represented in magenta, cyan, and green colors. The solvent molecules were omitted for clarity. (b) Hydrogen bond interactions between the Shh pseudo-active site (magenta) and Hhip L2 (green) are shown in black dotted lines and the hydrogen bonding residues are displayed as stick model. (c) The result of multiple sequence alignment between Hhip L2 and various species of Ptchs [21].

Cohesion of the ligand protein, Shh, to its receptor, Ptch, is an essential step in the activation of the Shh signaling pathway. In this interaction, the importance of PL2 was previously reported through several experiments by deleting the PL2 [15], [19], [20]. Our research aimed at the development of Shh inhibitor candidates using molecular dynamics and structure-based drug design approaches. For the drug design work, 3D structural information of the target was necessary. The structure of Shh was extracted from a crystal structure complex between human ShhN and human Hhip (PDB ID: 3HO5) and water molecules were removed. The crystal structure of Shh-Hhip was determined at a resolution of 3.01 Å containing one zinc and two calcium ions at the pseudo-active site of Shh (Fig. 1a and b) [21]. We attempted to investigate the conformational changes of the Shh induced by its receptor binding. However, x-ray predicted 3D structure of the Ptch is not available. We endeavored, vicariously, to model the key loop peptides of Ptch based on the 3D structure of homologous loop. In order to identify the conserved amino acid sequences between various species of Ptchs and the Hhip L2, a multiple sequence alignment of them reported by Ivan Bosanac *et al.* [15] was reproduced using *ClustalX2* program (Fig. 1c) [22]. Modeling of PL2 was possible based on the Hhip L2 template structure using *Build and Edit Protein* module of *Accelrys Discovery Studio 2.5.5* (DS) program [23].

Utilization of MD simulation has become a common tool in computing physical motions of biological molecules as a function of time and easier to explain the properties of a model system through simulations than experiments on a genuine system [24]. To compare the molecular behaviors between the model of human PL2 and human Hhip L2, the region made up of M373-D387 of Hhip L2 was extracted from the human Shh-Hhip complex structure. Additionally, in order to refine the fragments of PL2 and Hhip L2 and also to observe the interactions with the Shh, a set of 2 nanoseconds (ns) MD simulations were performed for each system with *GROMOS96 43a1* force field using *GROMACS 4.5.3* program. Hydrogen atoms were added to each protein complex structure and structural problems of all proteins such as incompletion of residues, nonstandard atom orders, nonstandard names, connectivity and bond orders, and unrefined termini (the N- and C-termini) were corrected through *Clean Protein* tool as available in DS. All the complex structures were immersed in cubic box which is filled with Simple Point Charge (SPC) explicit water models [25]. Charge neutralization of the complex structures was done by adding four chlorine ions in the water box. The systems of Shh-Hhip L2 and Shh-PL2 were minimized with maximum 10000 steps and energy tolerance of 2000 kcal·mol^−1^·nm^−1^ using steepest descent method. During the energy minimization and further simulations, only the atoms in its neighbor list with a cut-off distance of 0.9 nm were calculated for the short-range potentials. Criterion distances for calculating the electrostatic and van der Waals forces in a short-range were assigned to 0.9 nm and 1.4 nm, respectively. Additionally, the Particle Mesh Ewald (PME) method [26] was employed for long-range electrostatic calculations. All simulations were performed under Periodic Boundary Conditions (PBC) [27] in all directions to simulate “infinite” periodic boundary conditions instead of a finite size. Simulations of equilibration in the systems were conducted continually under conditions that all types of bonds are constrained using the Linear Constraint Solver (LINCS) algorithm [28] but the SPC water models and the chlorine ions are allowed to move freely for 100 picoseconds (ps) at a constant temperature of 300 K and pressure of 1 bar. Finally production simulations of 4 ns for each system were performed under unrestrained conditions and the same constant temperature and pressure with the preceding equilibrium simulations. During production simulations, the atomic coordinates of each system were updated every 1 ps. For an analysis of different binding modes of Hhip L2 and PL2 against the Shh pseudo-active site, the final snapshot of the Shh-Hhip L2 was superimposed to Shh-PL2 using *Superimpose Proteins* module in DS.

### Verification of roles of the metal ions

A series of 2 ns MD simulations of the Shh-PL2 with different compositions of the metal ions were conducted in order to identify the fact that the metal ions coordinated in the Shh pseudo-active site have a pivotal role in binding with its receptors or antagonists [17], [29]. Prior to MD simulations, we designed several types of Shh-PL2 complexes that contained only the zinc ion (without the two calcium ions), only two calcium ions (without the zinc ion), and without any ions in the complex structure. The starting structure of this process was the final snapshot of the 2 ns MD simulation of the Shh-PL2 complex. Upon the 2 ns MD simulations, final snapshots of the MD simulations with different metal ion compositions were superimposed including one of the preceding MD simulations of Shh-PL2 containing all metal ions through the *Superimpose Proteins* module in DS.

### Preparation of protein-inhibitor complex structure

The same procedure of the MD simulations of the Shh-PL2 was extended to 4 ns. Additionally, the conformational changes of the Shh induced by an inhibitor binding were also investigated through MD simulation. In order to obtain the Shh-robotnikinin complex structure, protein-ligand docking simulation was conducted using *LigandFit* module of DS. A 2D structure of robotnikinin was built using *ChemSketch 12* program [30] and it was converted into 3D structure using DS. A ligand binding site was defined at the Shh pseudo-active site which interacts with the Hhip L2 or the PL2.

In the process of docking simulations, diverse ligand conformations were generated using Monte Carlo algorithm by randomizing the torsion angles while bond lengths and bond angles are unaffected. The generated ligand conformations were energy minimized with *CHARMm* force field and a gradient tolerance of 0.001 using *Smart Minimizer* option to ensure correct bond lengths and bond angles of the conformations. In terms of energy minimization, steepest descent method up to 1,000 steps followed by conjugate gradient method was utilized until the energy of the conformations converged to a local minimum. During the final step of docking using *LigandFit* program, all the minimized conformations compared with the shape of the Shh pseudo-active site and redundant conformations were rejected in the meantime. The degree of fitness of each docked pose was evaluated using multiple scoring functions (LigScore1, LigScore2, -PLP1, -PLP2, Jain, -PMF, Ludi 1, Ludi 2, Ludi 3, -PMF04, DOCK SCORE). The multiple scores resulted from *LigandFit* docking calculations for each docked pose were assessed by consensus scoring and prioritized by descending order using *Consensus Score* module of DS. The docked pose of robotnikinin identified with top consensus score was selected as the favorable conformation and the Shh-robotnikinin complex structure was prepared.

Prior to the MD simulation of the Shh-robotnikinin complex, the charges for the charge groups of robotnikinin and its atomic coordinates and topologies were generated in suitable format for *GROMOS96 43a1* force field using the *PRODRG 2.5* server [31]. Finally 4 ns MD simulation of the Shh-robotnikinin complex structure was performed.

### Conformation clustering

In order to find each highly populated cluster from the all conformations of the Shh generated from the systems of the Shh-PL2 and Shh-robotnikinin, clustering method of *GROMACS 4.5.3* program was employed. Root-mean-square deviation (RMSD) criteria of clustering for conformations of the Shh backbone in the two systems were set to 0.05805 and 0.0587 nm, respectively. A conformation was classified to a cluster when its distance to any element of the cluster is less than its criteria. From each highly populated cluster, representative structure that is structurally close to the middle structure of the cluster was calculated.

### Pharmacophore modeling

Pharmacophore modeling studies were done using the representative structures obtained from the two independent MD simulations and clustering. In the Shh-PL2 complex structure, intermolecular hydrogen bonding residues of the Shh (K87, T125, E176, H180) and zinc ion and one of the calcium ions coordinated with the aspartate residue present in the edge of PL2 were considered as the essential interacting points for the generation of pharmacophoric features. To calculate chemical features of the Shh, PL2 structure was removed from the complex and subsequently the chemical features complimenting the essential components of the pseudo-active site were generated within the sphere diameter of 24 Å located in the center of the intermolecular hydrogen bonding residues and the metal ions using *Interaction Generation* module of DS. A generation of chemical features based on the Shh-robotnikinin complex structure was also performed in the same manner.

### Searching of drug-like molecules

Virtual screening procedures were performed using the generated pharmacophore models as 3D structural queries to retrieve compounds from the synthetic small molecule library, named ASINEX. A structural significance of these two pharmacophore models is the structural complementarity against the conformations of Shh pseudo-active sites induced upon the binding of PL2 or robotnikinin. The *Ligand Pharmacophore Mapping* module of DS was used with following options: *Best Mapping Only*: true, *Maximum Omitted Features*: 0, *Fitting Method*: flexible. Pharmacophore mappings of the robotnikinin against each of the two pharmacophore models were also required to calculate the fit values of them and to set the fit value as criterion for selecting the compounds that have relatively higher complementarities from the results of the pharmacophore mapping of the database. The mapped conformations of the hit compounds resulted from the two pharmacophore mapping calculations were selected based on the fit value of robotnikinin. All the compounds retrieved with the fit values higher than that of robotnikinin were selected and used in the next step of study. Evaluation of drug-likeness of the mapped compounds were facilitated by employing the Lipinski's rule of five and ADMET (Absorption, Distribution, Metabolism, Elimination and Toxicology) filtrations using *Lipinski Filter* and *ADMET Descriptors* modules of DS.

### Molecular docking and consensus scoring

Binding modes and fitness of the filtered compounds at the pseudo-active site of Shh were calculated using the *LigandFit* module of DS with the same parameters used in the docking of robtnikinin. Each of the two representative structures of Shh resulted from the previous clustering procedures was utilized as a receptor protein. In order to obtain more refined binding site, the filtered compounds with the high fit value from each of the pharmacophore mapping calculations were used in initial docking procedure and the refined binding sites were determined based on the docked modes. The *Consensus Score* module of DS was employed to prioritize the docked compounds on the basis of the calculated values through multiple scoring functions.

## Results and Discussion

### Role of the PL2 and significance of the metal ions in the ligand-receptor binding

The results of the multiple sequence alignment between the various species of PL2s and the human Hhip L2 showed the fact that the glycine residue was mutually identical and hydrophobic residues were similarly encompassed (Fig. 1c). The L2-like loops of various species of Ptch receptors were made up of more hydrophobic residues compared with Hhip L2. The E381 and D383 residues of Hhip L2 were conserved with chemically similar types of amino acids. In the 3D structure of Hhip L2, D383 coordinated with the zinc ion while E381 closely neighbored to one of the two calcium ions (Fig. 1b).

**Figure 2:**
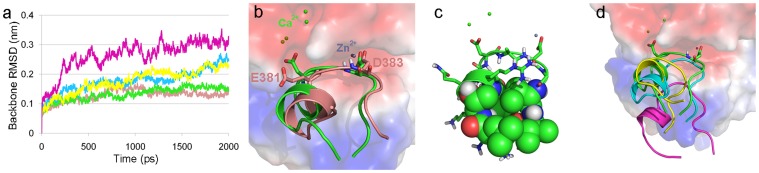
Comparison of the binding of loop peptides at the pseudo-active site of Shh. (a) RMSD plots for Shh backbone during 2 ns MD simulations for following complex structures: Shh-Hhip L2 complex with all metal ions, salmon; Shh-PL2 with all metal ions, green; Shh-PL2 with no zinc ion, yellow; Shh-PL2 with no calcium ions, cyan; Shh-PL2 with no metal ions, magenta. All loop structures also painted according to the colors of RMSD plots. (b) Superimposition of final snapshots of the Shh-Hhip L2 and Shh-PL2 complexes. Electrostatic potential surface was calculated from the Shh of Shh-PL2 structure. The zinc and calcium ions are shown as bluish-gray and green spheres. (c) The PL2 forming hydrophobic residues are shown by the space-filling model. (d) Superimposition of all complex structures of the Shh-PL2 with different composition of the metal ions. Electrostatic potential surface was calculated from the Shh of Shh-PL2 structure.

In order to study the function of human Ptch1 L2-like loop against human Shh pseudo-active site, a structure of human Ptch1 L2-like loop (PL2) was modeled based on the 3D structure of Hhip L2 in place of a full-length Ptch structure and this model was refined in the Shh-bound state through 2 ns MD simulations. For the comparison of the molecular behavior of PL2 with the Hhip L2 against the Shh, 2 ns MD simulation of the Shh-Hhip L2 was also performed as a control system. A validity of these two MD simulations was evaluated through RMSD calculations of each Shh backbone (Fig. 2a). The RMSD plots of both simulations were converged to plateaus and sustained in the near-native state. Final snapshots resulted from these two MD simulations were superimposed and binding modes of Hhip L2 and PL2 at the Shh pseudo-active site were compared (Fig. 2b). The final snapshot of Shh-PL2 showed different interaction mode to a certain degree in contrast to that of the Shh-Hhip L2. The D383 of Hhip L2 interacted with the zinc ion and the E381 of Hhip L2 located nearby a calcium ion but not coordinated with it directly. On the other hand, an aspartic acid of the PL2 corresponding to the E381 of Hhip L2 has coordinated with the calcium ion while simultaneously another aspartic acid of the PL2 corresponding to the D383 of Hhip L2 anchored to the zinc ion. The noose shape of PL2 was maintained during the MD simulations presumably due to the interactions of hydrophobic residues that consists of two isoleucines, two leucines, and one threonine of the PL2 corresponding to the I374, T375, M379, L385, and S386 of Hhip L2 (Fig. 2c).

The importance of the metal ions in binding between the Shh and PL2 was verified through 2 ns MD simulations with following conditions: Shh-PL2 with only zinc ion, with only two calcium ions, or without any ions. Prior to the several MD simulations, the final snapshot structurally adjusted from the 2 ns MD simulation of Shh-PL2 was used as the initial structure for each MD simulation because the final snapshot was stabilized by its structural adjustment. After a series of MD simulations, backbone RMSD values of each Shh complex were calculated to compare the structural changes (Fig. 2a). The Shh structure in both the zinc and calcium ions present complex systems has deviated much from its initial conformations meanwhile in case of the system with no ions the structure was severely affected. These observations were contrastive with respect to the result of Shh-PL2 simulation which contained all metal ions. In order to investigate a degree of separation of each L2-like loop, positions of PL2s were compared by superimposing their final snapshots (Fig. 2d). The removal of the calcium ions or zinc ion were resulted in slight loop drifts whereas the non-ion system led to the large-scale separation of the L2-like loop from the Shh pseudo-active site and these results were in accordance with the results of the RMSD calculations.

Taken together, we concluded that the metal ions coordinated in Shh have a pivotal role in the binding interaction of Shh with PL2. We hypothesized that introduction of the physical disturbance using a potent inhibitor with high affinity to the Shh pseudo-active site that holds the three metal ions can be a valuable expedient for interrupting the Shh signaling pathway.

### Cluster analysis for conformations of target protein

A clustering method was employed with an aim of finding representative Shh structures from the highly populated regions of the MD simulation results of the Shh-PL2 and Shh-robotnikinin complexes. To obtain a complex structure of the Shh with robotnikinin prior to MD simulation, docking simulations and consensus scoring calculations were conducted. The best binding pose of robotnikinin at the Shh pseudo-active site was selected based on its consensus score and interactions with the metal ions as well as the pseudo-active site residues (data not shown). The 4 ns MD simulations of Shh-robotnikinin complex were conducted and in order to accomplish the time consistency, the production simulation of Shh-PL2 complex was extended to 4 ns.

**Figure 3:**
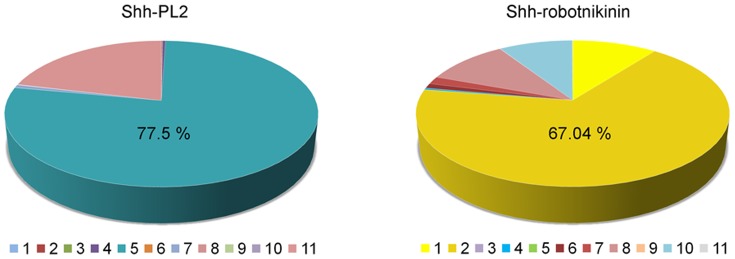
Pie charts of the clusters for backbone conformations of the Shh. (a) The highly populated cluster (cluster 5) of Shh-PL2 is colored in slashdot green. (b) The highly populated cluster (cluster 2) of the Shh-robotnikinin is colored in gold.

Each set of all conformations generated from these two MD simulations were clustered with the specific criterion RMSD of the Shh backbone structure and the total conformations of the systems were sampled into 11 clusters. The cluster 5 and cluster 2 of Shh-PL2 and Shh-robotnikinin complexes have accounted 77.5% and 67.03% of the conformations generated from each MD simulation (Fig. 3). We concluded from this result that each Shh was maintained in a certain conformational state while binding with PL2 and robotnikinin. From each cluster, the representative structure that is structurally close to the middle structure of the cluster was calculated. For the Shh-PL2 and Shh-robotnikinin systems, snapshots at 2663 ps and 2658 ps were selected as representative structures, respectively.

### Dynamic Shh-based pharmacophore modeling

**Figure 4:**
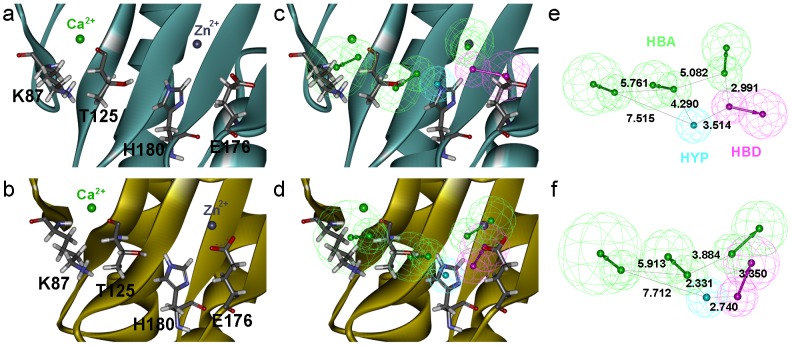
Design of structure-based pharmacophore models. The metal ions and key residues of the representative Shh structure of (a) the Shh-PL2 (slashdot green) and (b) the Shh-robotnikinin (gold). The zinc and calcium ions are marked by blue-gray and green spheres, and the key residues are represented by stick model. Chemical features are color coded as follows: HBA, green; HBD, magenta; HYP, cyan. Selected chemical features based on the representative Shh structure generated from (c) the Shh-PL2 and (d) Shh-robotnikinin. Completed pharmacophore models of (e) the Shh-PL2 and (f) Shh-robotnikinin.

To retrieve hit compounds of diverse chemical scaffolds from a chemical compound library, the generated pharmacophore models with shape and chemical complementarities to the representative structures of Shh-PL2 and Shh-robotnikinin complex were created. These dynamic structure-based pharmacophore models that are reflecting critical conformations of the Shh binding with the PL2 or the robotnikinin are of much significance because the single static structure cannot explain the dynamic character of a target protein [32]. After removing the PL2 or robotnikinin from each representative structure complex, diverse chemical features were generated within a calculating range of 24 Å centered on the pivot point of the metal ions and key residues (K87,T125,E175,H180) of Shh (Fig. 4a and b). Total numbers of chemical features generated from the representative structures of Shh-PL2 and Shh-robotnikinin were 623 and 661, respectively. Chemical features that constitute the final pharmacophore models were selected based on the metal ions and key residues (Fig. 4c and d). Both the pharmacophore models were made up of five chemical features which consists of three hydrogen bond acceptors (HBA), one hydrogen bond donor (HBD), and one hydrophobic (HYP) chemical features (Fig. 4e and f). The pharmacophore models developed from Shh-PL2 and Shh-robotnikinin complexes were named as Pharm-P and Pharm-R, respectively. The zinc ion and T125 corresponded to the two HBAs, and the HBD and HYP were created against the E176 and H180 residues in both of the two representative structures. The other HBA was the averaged chemical feature to interact with one of the two calcium ions and K87. This averaged feature was generated from the two overlapping HBA features originated against these two components (one calcium ion and K87). Comparing the pharmacophore models, the 3D positions, orientations of the chemical features, and inter-chemical feature distances were different particularly for the HBD corresponding to E176. This difference in the pharmacophore models explains the dynamic behavior of the Shh structure upon binding of diverse molecules (PL2 and robotnikinin).

### Drug-like compounds searching

**Figure 5:**
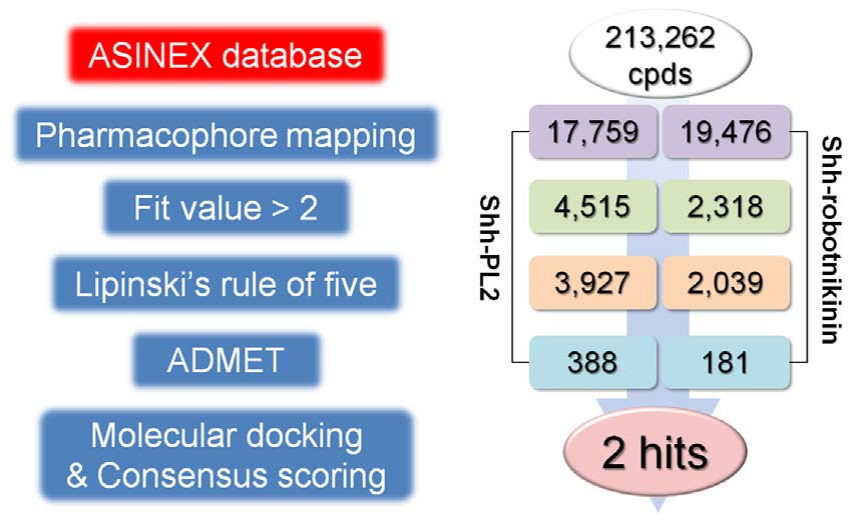
Flow chart of virtual screening procedure used in the study.

A series of virtual screenings were performed against the ASINEX database which consists of a total number of 213,262 diverse chemical compounds (Fig. 5). The first step of virtual screening procedure was the pharmacophore mapping calculation using the pharmacophore models generated from the representative structures of Shh-PL2 and Shh-robotnikinin complexes. The pharmacophore mapping calculations have resulted in 17,759 and 19,476 hit compounds for the Pharm-P and Pharm-R, respectively.

In order to check the validity of Pharm-R and to measure a fit value of a known inhibitor, a pharmacophore mapping calculation for the robotnikinin was performed. The mapping resulted in a fit value of 1.89 and based on this fit value cut-off fit value 2 was fixed to filter the mapped database hit compounds. From the results, it was found that robotnikinin has only mapped onto Pharm-R but not Pharm-P. The minimum fit value 2 was also fixed as a cut-off value to filter the mapped compounds retrieved through the Pharm-P model. The numbers of obtained compounds after fit value filtration for the Shh-PL2 and Shh-robotnikinin were 4,515 and 2,318, respectively.

Drug-like properties of the mapped compounds were assessed through the Lipinski's rule of five in order to exclude unnecessary molecules. The mapped compounds that satisfy the following rules were selected as drug-like compounds (i) less than 5 hydrogen bond donors, (ii) not more than 10 hydrogen bond acceptors, (iii) molecular weight not greater than 500, and (iv) logP value less than 5 [33]. Drug-like compounds of 3,927 and 2,039 were retrieved from the mapped compounds through the Pharm-P and Pharm-R models. The potential toxicities of these drug-like compounds also were evaluated through estimating their ADMET properties. Potentially toxic compounds were filtered out from the list of drug-like molecules if they disobey the following properties (i) good or moderate human intestinal absorption, (ii) low blood brain barrier (BBB) penetration, (iii) no inhibition of CYP2D6, and (iv) no hepatotoxicity. The ADMET filtration resulted in the potentially nontoxic compounds of 388 and 181 from the drug-like compounds retrieved for the Pharm-P and Pharm-R, respectively.

### Molecular docking and consensus scoring

Protein-ligand docking simulation was carried out to select hit compounds with high binding affinity to the Shh pseudo-active site and to investigate the binding modes of hit compounds identified through the Shh-PL2 and Shh-robotnikinin complexes. A designation of binding site was a prerequisite for the docking simulations therefore the pseudo-active sites of Shh protein of Shh-PL2 and Shh-robotnikinin complexes were selected as binding sites. To acquire detailed binding site, initial docking simulations at each pseudo-active site were performed only with the potentially nontoxic compounds scored highest fit values. In case of the Pharm-P, a hit compound named BAS 13382303 has shown the highest fit value of 3.91 whereas in case of the Pharm-R, another hit compound BAS 03200101 has shown the highest fit value of 4.02. More specified binding sites of the two pseudo-active sites were appointed based on the binding modes of the compounds of high fit values.

**Figure 6:**
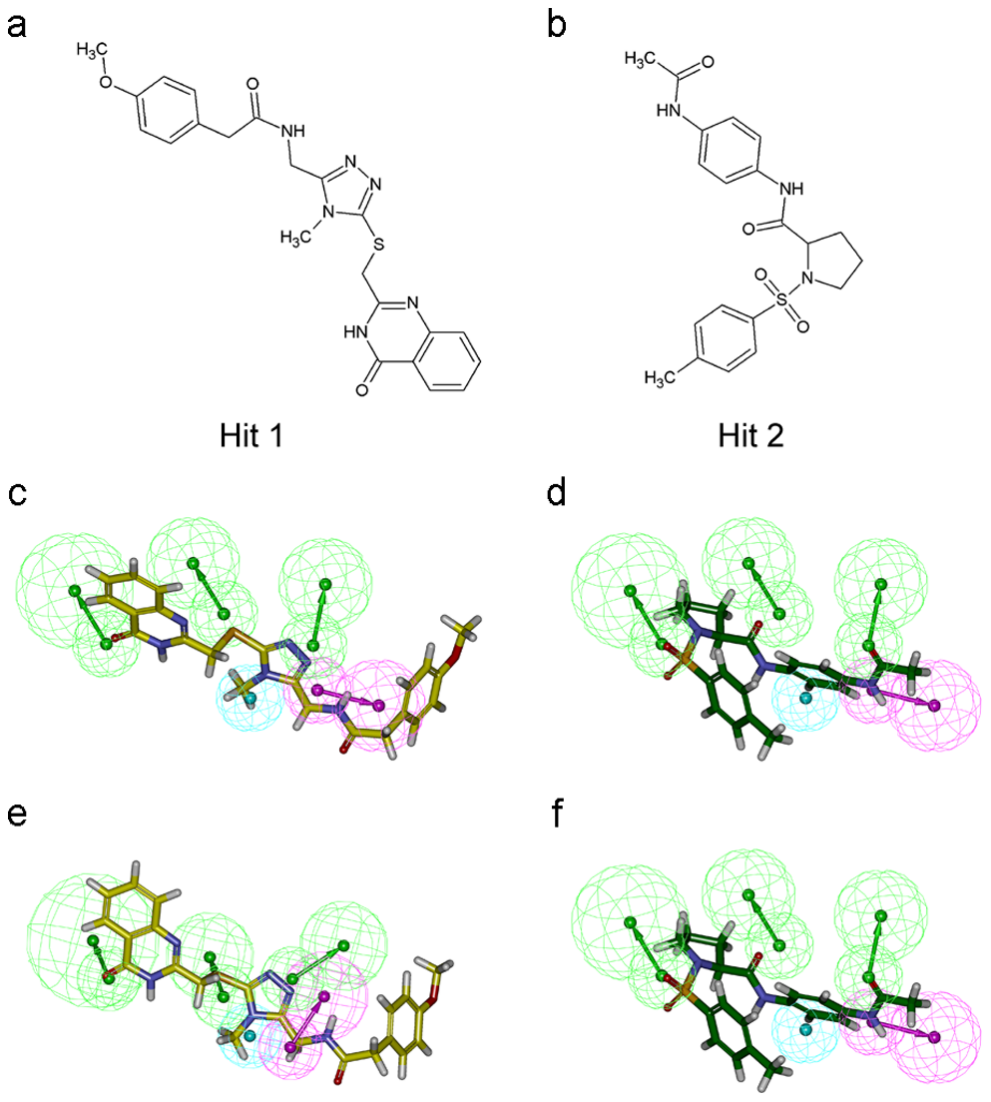
Final hit compounds and their pharmacophore overlay. The hit compounds depicted in stick representation. 2D chemical structures of (a) Hit 1 (BAS 13382537) and (b) Hit 2 (BAS 06350510). Mapping of (c) Hit 1 and (d) Hit 2 upon Pharm-P. Mapping of (e) Hit 1 and (f) Hit 2 upon Pharm-R.

Large-scale docking simulations were executed with the purpose of distinguishing the binding affinity of potential hit compounds at each pseudo-active site through the multiple scoring functions of 11 types. The docking simulations of all potentially nontoxic compounds at the pseudo-active sites of Shh-PL2 and Shh-robotnikinin complex resulted in 3,804 and 1,808 docked poses, respectively. The consensus scoring function was used to align all docked poses in descending order considering all calculated values. In the results of the consensus scoring calculations, we analyzed and selected only the compounds with high consensus scores. A total of 92 poses of 49 different compounds and 16 poses of 14 different compounds were obtained from the pseudo-active sites of Shh obtained from Shh-PL2 and Shh-robotnikinin complexes and used in molecular docking. Our aim of this procedure was to find the hit compounds with high affinity for both of the Shh pseudo-active site of representative structures of Shh-PL2 and Shh-robotnikinin complexes. The overlapping hit compounds were searched from the highest consensus scoring compounds and eventually 8 docked poses of two different compounds, namely, BAS 13382537 (Hit 1) and BAS 06350510 (Hit 2), were obtained (Table 1, Fig. 6a and b). The Hit 1 was mapped against the Pharm-P model with fit value of 2.42, and the fit value of the Hit 2 on the same model was 3.59 (Fig. 6c and d). In case of Pharm-R model, the Hit 1 was mapped on the model with the fit value of 2.78 and the Hit 2 mapped with the fit value of 2.43 (Fig. 6e and f).

**Table 1:** Overlapping binding modes of the hit compounds between the Shh-PL2 and Shh-robotnikinin derived pharmacophore models.

Scoring functions	Shh-PL2						Shh-robotnikinin	
	Hit 1				Hit 2		Hit 1	Hit 2
	pose 1	pose 2*	pose 7	pose 9	pose 5*	pose 6	pose 7*	pose 1*
LigScore1	3.35	3.52	4.67	4.61	2.7	3.08	2.82	3.07
LigScore2	4.38	4.18	5.12	5.01	4	4.79	3.88	3.71
-PLP1	110.4	111.16	103.79	103.04	93.68	91.15	90.07	84.06
-PLP2	93.84	90.55	89.4	88.99	89.92	87.6	77.29	75.47
Jain	1.98	3.23	2.08	2.48	1.52	1.93	3.62	1.29
-PMF	191.87	162.71	162.67	168.4	165.44	160.06	112.26	115.69
Ludi 1	297	345	402	422	404	378	382	299
Ludi 2	315	344	350	341	396	366	358	320
Ludi 3	489	596	535	528	652	550	484	403
-PMF04	153.49	141.28	131.69	135.74	132.67	135.09	104.03	102.27
DOCK SCORE	105.528	104.534	99.023	98.821	87.448	86.675	81.975	80.457
Consensus	11	11	11	11	11	11	11	11

* Docking poses were selected based on interactions with the metal ions and key residues of Shh for further binding mode analysis.

**Figure 7:**
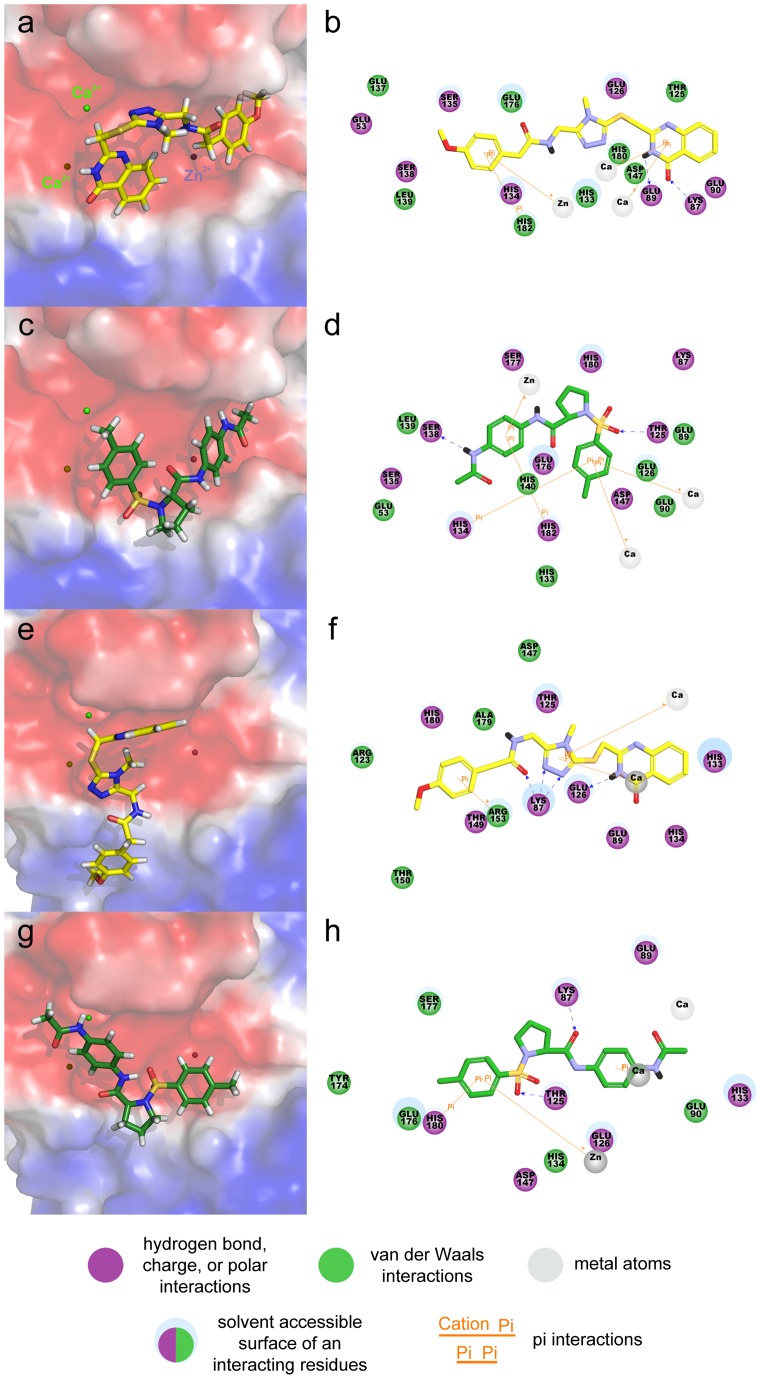
Comparison of binding modes of the hit compounds. Hit 1 and Hit 2 are depicted in yellow and green stick representations. Electrostatic potential surfaces were calculated from representative Shh structures of (a,c) the Shh-PL2 and (e,g) Shh-robotnikinin. 2D diagrams for interactions of hit compounds with the representative Shh structures of (b,d) the Shh-PL2 and (f,h) Shh-robotnikinin. Blue dashed lines indicate hydrogen bonding interactions between protein and ligand.

Finally, we chose the best docked poses of the common hit compounds based on interactions with the metal ions and key residues of each Shh pseudo-active site of the Shh-PL2 and Shh-robotnikinin complexes. In case of Hit 1 binding to the representative structure of Shh-PL2, the phenyl ring of methoxyphenyl group formed a pi-pi interaction with H182 and a cation-pi interaction with the zinc ion (Fig. 7a and b). The dihydroquinazolinyl group associated with cation-pi interactions to the two calcium ions, and hydrogen bonds were detected through its carbonyl and fused amino groups with the K87 and E89, respectively. The docked Hit 2 at the Shh pseudo-active site of Shh-PL2 complex has formed hydrogen bond interactions through its acetamide and sulfonyl groups with S138 and T125 residues, respectively (Fig. 7c and d). The phenyl ring of toulene moiety formed a strong pi-pi interaction with the H134 and cation-pi interactions with two calcium ions present in the pseudo-active site. The other phenyl ring interacted by forming a pi-pi interaction with the H182 and cation-pi interaction with the zinc ion. In case of the Hit 1 bound to the Shh-robtnikinin complex, the carbonyl part of amide group and the two fused adjacent nitrogen atoms of five-membered triazole ring have formed hydrogen bonds with K87 (Fig. 7e and f). Additionally, the fused amino group next to carbonyl part of quinazoline ring has interacted with E126 through a hydrogen bond. Two cation-pi interactions were detected between the phenyl ring of methoxyphenyl and R153 as well as between the triazolyl ring and each of the two calcium ions. The binding mode of Hit 2 at the Shh pseudo-active site of Shh-robotnikinin complex has also showed cation-pi interactions between the phenyl ring attached to the acetamide moiety and one of the two calcium ions as well as between the phenyl ring of toluene group and the zinc ion (Fig. 7g and h). The phenyl ring of this toluene also exhibited a pi-pi interaction with H180. Hydrogen bonds were formed through the sulfonyl and central amino groups with the T125 and K87 residues.

Taken together, the Hit 1 and 2 were associated with the strong molecular interactions to the each Shh pseudo-active sites. These strong interactions of the two hit compounds presumably can interrupt the Shh signaling transduction, because the binding of the PL2 at the Shh pseudo-active site is the essential step in the signaling transduction process.

## Conclusion

In this study, we have investigated the Shh-PL2 and Shh-robtnikinin complexes to obtain deep molecular insight of specific interactions between the Shh and Ptch proteins. We confirmed that the metal ions present in the Shh pseudo-active site were essential components in maintaining the direct binding between the PL2 and Shh through the PL2 modeling and a series of MD simulations with different metal ion compositions. Therefore, the metal ions are the key factors for the binding of PL2 with Shh. The residues of Shh that enable the hydrogen bonds with PL2 that is homologous to the Hhip L2 were also chosen as the key factors. In structure-based drug design procedure, the utilization of the dynamic conformational changes of a target protein upon receptor or small molecule binding is important because a single stationary structure is not sufficient for elucidating detailed structural rearrangement effected by the inhibitor. We used robotnikinin as the known inhibitor which has inhibitory effect on the Shh signaling transduction and a MD simulation was introduced for generating conformations of the Shh-robotnikinin complex structure as a function of time. Subsequently, a dynamic target-based pharmacophore model (Pharm-R) which is complementary to the pseudo-active site of the inhibitor-induced Shh was created.

We devised a novel concept that is generating a dynamic target-based pharmaocphore model with consideration of conformational changes of a target-receptor complex structure. As the 3D structure of Shh receptor protein Ptch was not solved yet, we employed PL2 model built based on the loops present in homologous proteins in our study instead of a full-length structure of Ptch. Another pharmacophore model (Pharm-P) was created based on the Shh pseudo-active site of representative structure extracted from the highly-populated region of MD simulations using clustering.

A series of virtual screening including ligand pharmacohpore mapping followed by drug-likeness and toxicity prediction were performed using the pharmacophore models (Pharm-R, Pharm-P) generated using the Shh-robotnikinin and Shh-PL2 complexes. Using the compounds filtered from the virtual screening, protein-ligand docking simulations and consensus scoring calculations were performed to find hit compounds with high affinity against both of the Shh pseudo-active sites. The binding affinities of the virtually screened compounds were calculated by the multiple scoring functions. These multiple scores were comprehensively evaluated through consensus scoring. Finally, we obtained two hit compounds (BAS 13382537, BAS 06350510) showing strong interactions with the key structural components of pseudo-active sites of both the Shh-PL2 and Shh-robotnikinin complexes.

Both of the hit compounds were found to accommodate well at the inhibitor-induced and receptor loop peptide-induced Shh structures with strong molecular interactions. The strong interactions of the hit compounds with the inhibitor-induced Shh structure may signify that the hit compounds have properties of an inhibitor with respect to the 3D structure of the target protein. The strong interactions between the hit compounds and receptor loop peptide-induced Shh structure may mean that the hit compounds possess the properties to bind the Shh pseudo-active site and thereby interrupt the binding of Shh and Ptch proteins. From these results, we concluded that two hit compounds identified in this study are of great potential to be the inhibitor candidates for the Shh signaling pathway. The approach of drug design considering not only inhibitor-induced target structure but receptor-affected target structure can be utilized usefully in designing potent Shh signaling inhibitor candidates.
